# Impact of an Educational Intervention on Adherence to Minimum Laboratory Re-testing Intervals (MRTI) Among Internal Medicine Residents

**DOI:** 10.7759/cureus.108895

**Published:** 2026-05-15

**Authors:** Sekar Dineshbabu, Debasis Naik, Ramadoss Ramu

**Affiliations:** 1 Internal Medicine, Jawaharlal Institute of Postgraduate Medical Education and Research, Puducherry, IND; 2 General Surgery, Jawaharlal Institute of Postgraduate Medical Education and Research, Puducherry, IND

**Keywords:** before-and-after study, clinical laboratory tests, guideline adherence, medical education, quality improvement, quasi-experimental study

## Abstract

Background

Overuse of repeat laboratory testing contributes to increased healthcare costs and patient burden, particularly in teaching hospitals where test ordering is often trainee-driven. Minimum re-testing intervals (MRTI) have been proposed as a strategy to reduce unnecessary investigations, but adherence remains suboptimal.

Methods

We conducted a quasi-experimental before-and-after study in the Department of Medicine of a tertiary care teaching hospital in South India. A structured educational intervention incorporating MRTI guidance was delivered to internal medicine residents. Medical records of hospitalised patients with a stay of more than or equal to five days were reviewed during pre-intervention (November 2025) and post-intervention (January 2026) periods. The primary outcome was adherence to MRTI recommendations. Comparisons were made using the chi-square test, with results reported as absolute differences with 95% confidence intervals (CI).

Results

A total of 863 and 824 laboratory tests were analysed in the pre- and post-intervention phases, respectively. Overall adherence to MRTI improved from 58.9% to 65.9%, corresponding to an absolute increase of 7.0% (95% CI: 2.4% to 11.6%; χ² = 8.68, p = 0.003). Improvements were observed across most test categories, with the largest increase seen in complete blood count (CBC) testing (+28.3%). Significant gains were also noted for renal function tests, electrolytes, and calcium-magnesium panels. In contrast, liver function tests showed no statistically significant improvement. Total testing volume decreased modestly without a reduction in testing intensity per patient.

Conclusions

A brief, structured educational intervention significantly improved adherence to recommended laboratory re-testing intervals, particularly for frequently ordered investigations. These findings support the role of targeted educational strategies as a pragmatic and scalable approach to improving laboratory stewardship in resource-constrained teaching hospitals, although test-specific and system-level interventions may be required for sustained impact.

## Introduction

Overuse of laboratory investigations is a growing concern in clinical practice, leading to increased healthcare costs and greater use of hospital resources [1]. The American Board of Internal Medicine (ABIM) Choosing Wisely campaign urges doctors to avoid routine lab tests in stable hospitalised patients, yet physicians often ignore this due to habits and many other patient-related factors, like decreased time to attend to the patient and to reassure themselves [2]. Repeated phlebotomy during hospitalisation has been associated with an increased risk of hospital-acquired anaemia in certain patients [3].

Research suggests that 20-30% of inpatient tests are unnecessary, often repeated sooner than required [4,5]. This issue is more common in teaching hospitals, where residents frequently order daily tests such as complete blood count (CBC), liver function tests (LFTs), and electrolytes without a clear indication [6]. A key reason is limited awareness about minimum re-testing intervals (MRTI)--the minimum time before a test should be repeated, based on test properties and the clinical situation [7]. Other contributing factors include the patient’s condition, consultant and resident practices, delays in reporting, and fear of sample rejection [8].

A retrospective review of CBC requests among inpatients in the Medicine department at a tertiary care hospital showed variation in testing patterns with intern staffing availability. In January, 1,101 admissions had 1,636 hemograms (1.49 per patient), compared to 1,023 admissions and 1,220 hemograms in February (1.19 per patient), representing a 25% higher testing rate in January (p = 0.00013). Although daily admissions were similar, more tests were ordered per day in January. This difference may reflect variations in staffing, as intern availability was limited during the month of January due to delayed postings following the COVID-19 pandemic, with subsequent deployment of 18 interns in the month of February in the Medicine department. These findings suggest that increased staffing availability may be associated with higher test-ordering intensity.

Based on this, we conducted structured small-group teaching sessions to improve awareness and adherence to MRTI among residents. Educational interventions have been shown to reduce unnecessary testing, and we expected that improving MRTI awareness would promote more appropriate test use, reduce patient discomfort, and support cost-effective care [9,10].

This study aimed to assess the impact of an educational intervention on adherence to recommended MRTI for routine blood investigations among internal medicine residents in stable inpatients.

## Materials and methods

Study design and setting

This study was designed as a quasi-experimental before-and-after evaluation of an educational intervention. It was conducted in the Department of Medicine of Jawaharlal Institute of Postgraduate Medical Education and Research, a government-funded, 2200-bedded, tertiary care teaching hospital in South India. The hospital functions as a major referral centre for both urban and rural populations and manages a wide spectrum of acute and chronic medical conditions. The Department of Medicine handles more than 300 inpatients and operates through multiple clinical units staffed by faculty, senior residents, junior residents, and interns, reflecting a typical academic training environment where laboratory investigations are frequently resident-driven.

Study participants

All internal medicine residents posted in the department of medicine during the study period (November 2025 to January 2026) were considered eligible. Out of the total 72 residents, 16 residents were working in the intensive care unit (ICU) and other departments as a part of their clinical training, making the eligible number 56, out of which 48 provided written informed consent and participated in the educational intervention. Participants included residents across different levels of postgraduate training (first-, second-, and final-year), ensuring representation of varying clinical experience. The intervention was delivered unit-wise in small groups to maximise participation. As the intervention targeted group behaviour and laboratory ordering patterns were assessed using anonymised patient records, individual resident-level prescribing behaviour was not tracked.

Selection of medical records

Medical records were retrospectively reviewed at the time of discharge for patients admitted under the Department of Medicine during the pre-intervention (November 2025) and post-intervention (January 2026) periods. Eligible records included adult inpatients with a hospital stay of more than or equal to five days to allow sufficient observation of repeat laboratory testing patterns. A consecutive sampling strategy was employed, whereby all eligible discharge records during the study periods were screened and included. This approach was used to minimise selection bias and reflect routine clinical practice. Only discharged patients were included to enable complete assessment of laboratory testing throughout the hospital stay. Patients aged >65 years were excluded because older adults often have multiple comorbidities, frailty, and a higher likelihood of clinical and biochemical instability, which may necessitate more frequent testing independent of appropriateness. Similarly, patients in the ICU, those on haemodialysis, receiving anti-tubercular therapy, or undergoing cytotoxic chemotherapy require protocol-based or high-frequency monitoring, which would limit the applicability of standard MRTIs. These exclusions were intended to create a more homogeneous cohort of clinically stable inpatients in whom adherence to MRTI could be meaningfully assessed.

Outcome measures

The primary outcome was adherence to MRTI, defined at the level of individual laboratory tests. For each test, the time interval between two consecutive measurements was calculated. A repeat test was considered adherent if it was performed at or beyond the recommended minimum interval, and non-adherent if performed earlier than the specified interval without a documented clinical indication (e.g., clinical deterioration, new symptoms, or evolving laboratory abnormality). Each component of a laboratory panel (e.g., sodium and potassium within electrolytes) was analysed separately. Adherence was calculated as the proportion of repeat tests meeting MRTI criteria out of total repeat tests performed during the clinically stable phase.

Sample size calculation

We initially planned to include 100 patient records in both pre- and post-intervention phases. Based on the study by Krasowski et al., which reported significant reductions in laboratory utilisation following electronic medical record-based interventions, we assumed a 30% reduction in repeat testing [11]. Accordingly, a sample size of 208 observations (104 per group) was estimated (80% power, α=0.05). As each laboratory test was analysed individually, 100 records per phase were expected to provide adequate observations. However, due to logistical constraints, only 65 and 56 records were included in the pre- and post-intervention phases, respectively.

Ethics approval and study procedure

This study was approved by the Institutional Ethics Committee. Eligible case records were analysed retrospectively to assess laboratory testing practices during the clinically stable phase of hospitalisation. Clinical stability was independently determined by two faculty members (principal investigator and co-investigator) using predefined criteria. Inter-rater agreement was ensured through independent assessment followed by consensus discussion in discrepant cases. Although formal kappa statistics were not calculated, predefined objective criteria were used to minimise subjectivity.

Vital sign stability was defined as the absence of fever or a declining temperature trend, heart rate <100/min without new arrhythmia, stable blood pressure without vasopressor support, respiratory rate <20-22/min without distress, and oxygen saturation ≥94% on room air (or baseline for chronic lung disease). Haemodynamic stability required the absence of hypotension or shock, no requirement for inotropes or intravenous fluid resuscitation, urine output >0.5 mL/kg/hour, and preserved mentation without new-onset delirium. Laboratory stability was defined as the absence of worsening trends, including falling haemoglobin, rising creatinine, increasing leukocyte count, or electrolyte abnormalities requiring urgent correction. Only laboratory tests performed during this clinically stable period were evaluated for adherence to MRTI.

Laboratory variables

Routinely ordered investigations, including CBC, renal function tests (urea and creatinine), electrolytes (sodium, potassium, calcium, magnesium), and LFTs, were recorded. As test panels are frequently ordered together, individual components were analysed separately to more accurately capture total testing volume and enable meaningful comparison.

MRTI

MRTIs were defined based on published recommendations from Cadamuro et al. [7], Lang et al. [9], and van Walraven and Raymond [10]. These intervals were applied only to patients deemed clinically stable to ensure contextual appropriateness. The MRTI window was evaluated from the onset of clinical stability until discharge, thereby avoiding inappropriate restriction of testing during the acute phase of illness.

Education intervention

The education intervention was delivered to all medicine residents, unit-wise, in small groups for the residents who provided written consent for receiving the education intervention. The education intervention was given either before or after their clinical rounds, making it convenient for them to attend, as they did not need to come at a separate time for the small group discussion. A total of 7 sessions were conducted, each lasting for about 30 min to ensure maximum resident participation. The education intervention was structured as follows:

 Awareness

The session opened by comparing laboratory ordering patterns during the intern-deficient period with current practice, illustrating how staffing availability influenced test ordering. The rationale for MRTIs was then discussed: excessive phlebotomy contributes to hospital-acquired anaemia, over-testing raises the likelihood of false-positive results and cascading investigations, and routine daily testing increases costs without improving patient outcomes. Both the Choosing Wisely campaign and the Royal College of Pathologists endorse MRTIs to curb overuse, and structured education with feedback has demonstrated improved adherence to appropriate testing practices.

 Standardisation

The session emphasised that understanding and applying MRTIs is essential for appropriate laboratory use, patient safety, and cost-effective care. Participants were introduced to recommended intervals for commonly ordered tests, based on evidence from Cadamuro et al. (2017) [7], Lang et al. (2019) [9], and van Walraven and Raymond (2003) [10]. Repeat testing was advised only at recommended intervals unless earlier testing was clinically indicated.

The MRTI recommendations provided to the residents are summarized in Table 1.

**Table 1:** MRTI for the tests.

Investigation	Recommended Interval	Situation Requiring Early Testing
Complete blood count (CBC)	3-5 days	Acute bleeding, suspected sepsis, rapidly falling hemoglobin
Electrolytes (Na, K, Ca, Mg)	48-72 hrs	Acute illness, electrolyte imbalance, diuretic therapy, IV fluids, arrhythmia risk
Renal function tests (urea, creatinine)	48-72 hrs	Acute kidney injury, nephrotoxic drugs, volume depletion,
Liver function tests (LFT)	7-14 days	Acute hepatitis, suspected drug-induced liver injury, worsening jaundice

Clinical Reasoning

Case-based scenarios were discussed to reinforce the practical application of MRTIs at the bedside. In the first case, a 55-year-old man with stable pneumonia on intravenous antibiotics and a normal baseline hemogram, routine daily hemogram testing was deemed unnecessary, with repeat testing appropriate only after 3-5 days or earlier if clinical deterioration, bleeding, or sepsis progression occurred. In the second case, a 40-year-old woman with improving hepatitis A, daily LFT monitoring was considered unnecessary, with repeat testing appropriate at 7-14 days unless worsening jaundice, coagulopathy, or new complications arose. Additional examples drawn from patients under the residents' own care were also discussed.

Decision Pause

To further strengthen clinical reasoning and reduce habitual ordering, we introduced the concept of a “decision pause” before requesting any repeat laboratory test. Participants were encouraged to consciously step back and apply a simple mental checklist, “The 5-Question Rule”, before ordering a test. This checklist was designed to interrupt habitual ordering patterns and promote deliberate, value-based decision-making.

The five-question checklist had the following questions:

(1) Has the patient’s clinical condition changed? (2) Will the result change my management today? (3) Was the last result abnormal and unstable? (4) Am I ordering this out of habit rather than necessity? (5) Is the interval shorter than the SOP minimum?

We emphasised that if the answer to Questions 1-3 is NO and to Questions 4 and 5 is YES, the repeat test should not be ordered.

 Reinforcement

To help reinforce the learning after the session, the checklist and table showing MRTI were made into a short and simple PDF and were shared in the Medicine residents’ WhatsApp group. This would give residents an easy reference they can quickly look at during their daily clinical work. By keeping the “5-Question Rule” easily available, we hope to encourage better decision-making.

The medical education was given in multiple small groups over a period of two weeks after the pre-education data collection was complete. After the education intervention was provided to most residents, post-intervention data were collected, which measured the adherence rate to MRTI among patients admitted after the intervention was completed.

Statistical analysis

Categorical variables are presented as frequencies and percentages, while continuous variables are expressed as mean ± standard deviation (SD) or median with interquartile range (IQR), as appropriate. The primary outcome, adherence to MRTI, was compared between pre- and post-intervention periods using the chi-square (χ²) test. Results are reported with corresponding χ² values and degrees of freedom. Effect sizes were calculated as absolute differences in adherence proportions, along with 95% confidence intervals (CIs), to estimate the magnitude and precision of the observed effects. Test-specific adherence rates were analysed similarly using chi-square tests. To account for multiple comparisons across laboratory test categories, a Bonferroni correction was applied, yielding an adjusted significance threshold of p < 0.01. Statistical analysis was performed using IBM SPSS Statistics (version 22; IBM Corp., Armonk, NY).

## Results

Study population

**Figure 1:**
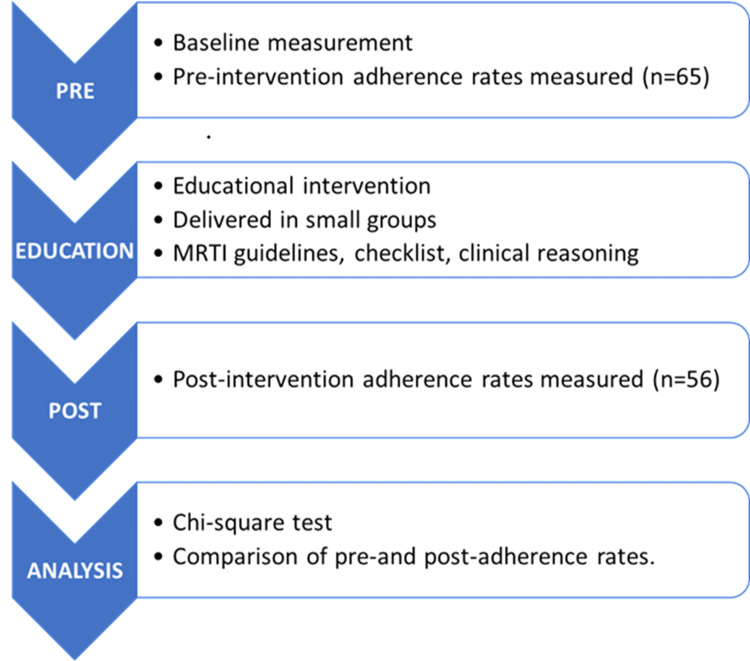
Study flow diagram

After applying inclusion and exclusion criteria, 65 and 56 case records were included in the pre- and post-intervention phases, respectively. The study flow diagram is shown in Figure 1.

Baseline characteristics of patients in the two groups were comparable. The mean age was 41.52 ± 12.35 years in the pre-intervention group and 44.16 ± 11.21 years post-intervention. The proportion of male patients was 46% (n=30) and 55% (n=31), respectively. Median length of hospital stay was 10 days (IQR 8-14) in the pre-intervention group and 11 days (IQR 9-14) in the post-intervention group.

Overall laboratory testing and adherence to MRTI

A total of 863 laboratory tests were analysed in the pre-intervention phase and 824 in the post-intervention phase, representing a modest reduction of 4.5% in overall testing volume.

In the pre-intervention phase, 508 of 863 tests (58.9%) adhered to MRTI, compared to 543 of 824 tests (65.9%) in the post-intervention phase. This corresponds to an absolute increase in adherence of 7.0% (95% CI: 2.4% to 11.6%), which was statistically significant (χ² = 8.68, df = 1, p = 0.003).

Conversely, non-adherent testing decreased from 41.1% to 34.1%, representing a relative reduction of 20.8%. When adjusted for patient-days, adherent testing increased by 12.8%, while non-adherent testing decreased by 16.4%, with no meaningful change in overall testing intensity.

Test-specific adherence

**Figure 2:**
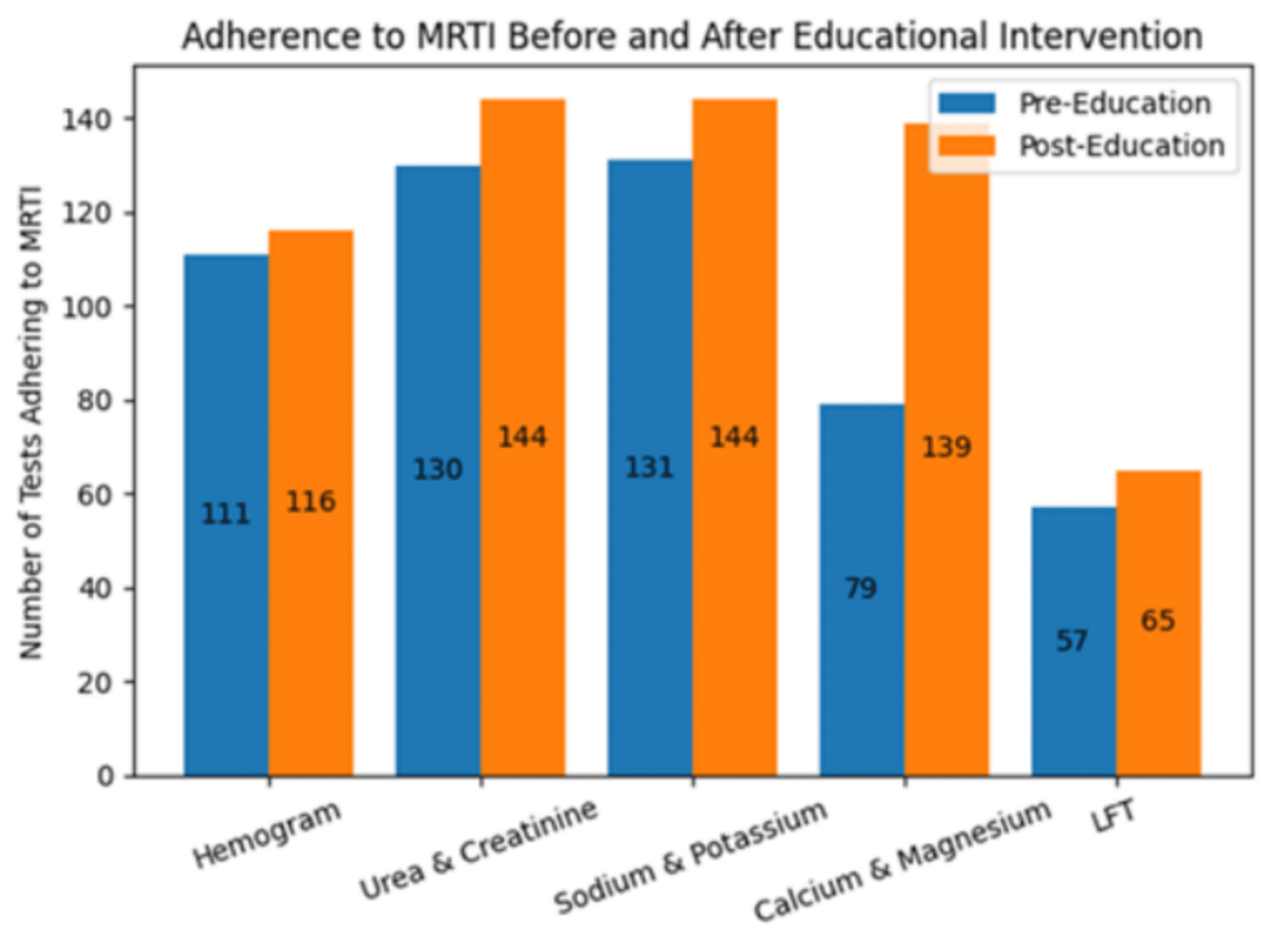
Number of tests done adhering to MRTI before and after education. MRTI: minimum re-testing interval

Following the educational intervention, adherence to MRTI improved across all categories of laboratory tests (Figure 2, Table 2).

**Table 2:** Adherence to MRTI before and after the educational intervention Footnotes: *n, number of tests adhering to MRTI; N, total number of tests done; MRTI, minimum re-testing interval

Test	Pre-education adherence % (n/N)^ *^	Post-education adherence % (n/N)^ *^	Absolute difference (%)	95%CI	χ² (df=1)	p-value
Hemogram	55.2 (111/201)	83.5 (116/139)	+28.3	19.9 to 36.7	30.5	0.0058
Urea and creatinine	59.4 (130/219)	74.2 (144/194)	+14.8	6.2 to 23.4	8.6	0.0035
Sodium and potassium	61.2 (131/214)	74.6 (144/193)	+13.4	4.7 to 22.1	8.3	0.0041
Calcium and magnesium	62.7 (79/126)	75.1 (139/185)	+12.4	3.6 to 21.2	12.2	< 0.001
Liver function tests	55.3 (57/103)	57.5 (65/113)	+2.2	-8.1 to 12.5	0.18	0.0012

The largest improvement was observed in hemogram testing, where adherence increased from 55.2% to 83.5% (absolute increase 28.3 percentage points, p = 0.0058). Improvements were also observed in renal function tests (59.4% to 74.2%, +14.8 percentage points, p = 0.0035), electrolytes (61.2% to 74.6%, +13.4 percentage points, p = 0.0041), and calcium and magnesium testing (62.7% to 75.1%, +12.4 percentage points, p < 0.001). Liver function tests showed a smaller improvement, from 55.3% to 57.5% (absolute increase 2.2 percentage points, p = 0.0012). After Bonferroni correction for multiple comparisons across the five test categories (adjusted significance threshold p < 0.01), all observed improvements were statistically significant for hemogram, renal function tests, electrolytes, and calcium-magnesium panels, while changes in liver function test adherence were not statistically significant.

## Discussion

This study demonstrates that a brief, structured educational intervention incorporating MRTI guidance was associated with a statistically significant improvement in adherence to recommended laboratory testing intervals in a tertiary care teaching hospital. Overall adherence increased from 58.9% to 65.9%, representing an absolute improvement of 7.0% (95% CI: 2.4% to 11.6%; χ² = 8.68, p = 0.003). This was accompanied by a reduction in non-adherent testing and a modest decline in total test utilisation, without a corresponding increase in length of hospital stay, suggesting that improved adherence did not compromise clinical monitoring.

A key finding of this study is the heterogeneous impact of the intervention across different laboratory tests. The largest improvement was observed in hemogram testing (+28.3%), followed by renal function tests and electrolyte panels (approximately +13 to +15%), and calcium-magnesium testing (+12.4%). These changes were statistically significant and remained robust after adjustment for multiple comparisons. In contrast, liver function tests showed only a small, non-significant improvement (+2.2%; 95% CI crossing zero), indicating that the intervention had a limited effect on this category of testing.

This differential response is clinically meaningful. Tests such as hemograms, renal function tests, and electrolytes are frequently ordered and are often subject to habitual or protocol-driven repetition, making them more amenable to behavioural interventions. In contrast, liver function testing may be more closely guided by dynamic clinical considerations, including drug monitoring and evolving disease states, which may justify more frequent reassessment. These findings suggest that while educational interventions can effectively address routine overuse, test-specific strategies may be required for investigations driven by greater clinical variability. 

The magnitude of improvement observed in this study is modest but important. An absolute increase of 7% in adherence represents a meaningful reduction in unnecessary testing when applied across high-volume inpatient settings. Given the low-cost and easily implementable nature of the intervention, such gains are likely to be scalable, particularly in resource-constrained teaching hospitals where laboratory overutilisation is common and often driven by trainee-led ordering practices. 

Educational interventions consistently reduce laboratory overutilisation, aligning with multicentre studies showing 10-30% drops in unnecessary tests via multi-modality approaches like lectures and feedback. For instance, a resident-focused program cut costs by promoting guidelines, reflecting our study’s focus on high-overuse tests like hemograms. Non-adherence to MRTI remains common (e.g., 4-14% for similar panels), with education proving cost-effective ($12,817 savings in one audit) [12].

The educational program worked well because it focused on the habit of ordering too many tests during hospital stays, especially blood counts and kidney function tests that are often repeated. Liver function tests improved only slightly (by 2.2%), likely because patients with liver problems were tested less frequently at baseline, which matched MRTI guidelines. The number of tests per patient decreased, the number of tests per patient-day remained comparable, indicating that the intensity of monitoring during hospitalisation was maintained and supporting the safety of the intervention [13].

These results also show that simple, low-cost educational programs can be an effective first step in improving laboratory test use, especially in resource-limited settings in India, where unnecessary testing increases costs and inpatient load is increasing every day. Adding MRTI alerts into electronic ordering systems can further improve results, as combined approaches have been shown to reduce unnecessary tests by 20-40% over time [14]. Involving trainees, as done in our study, also helps build a culture of responsible test ordering and supports the need for regular training during residency.

Although our study showed a positive effect of the educational intervention, it was limited by the simultaneous transition of the hospital information system to the Centre for Development of Advanced Computing (CDAC) platform. During this period, test ordering became more difficult, as reports were not easily available online. This may have independently reduced the number of tests ordered and influenced our results.

This finding highlights that residents’ test-ordering behaviour is strongly affected by system-related factors. Previous studies, including those by Krasowski and Bates, have shown that changes in electronic medical records and ordering systems can significantly influence how often tests are ordered [11,15,16]. When the process becomes less convenient, clinicians tend to be more careful and selective. Combining behavioural interventions with system-level strategies may be necessary for sustained improvement, as computerised alert systems based on MRTI have been shown to reduce inappropriate laboratory test requests [17].

In addition, inappropriate or excessive laboratory testing is a well-recognised issue in clinical practice [4]. Therefore, test-ordering behaviour is shaped not only by clinical judgment but also by how easy or difficult the system is to use. Future efforts to reduce unnecessary testing should combine education with system-level improvements in workflow and electronic platforms.

Strengths and limitations

Our study implemented a simple, low-cost educational intervention and evaluated its impact using objective laboratory ordering data. By including multiple commonly ordered laboratory tests, the findings provide a broader and more practical understanding of test-ordering practices.

Several methodological considerations should be noted. First, the study employed a before-and-after design without a concurrent control group, which limits causal inference and introduces the possibility of temporal confounding. Second, although consecutive sampling of eligible records was used to minimise selection bias, the relatively small sample size and single-centre setting may limit generalisability. Third, exclusions such as patients aged >65 years, those requiring intensive monitoring (e.g., ICU care, dialysis, chemotherapy), and prolonged or complex illness were applied to reduce clinical heterogeneity; however, this may restrict applicability to more complex patient populations. Fourth, adherence assessment was based on predefined MRTI criteria, and while efforts were made to standardise classification, some degree of subjective interpretation may persist. Fifth, our study did not assess the long-term sustainability of behaviour change. Finally, the transition to the CDAC system from the Hospital Information System (HIS) may also have influenced data capture and ordering patterns.

Despite these limitations, this study provides practical insights into improving laboratory utilisation in real-world clinical settings. It highlights that simple, targeted educational interventions can produce measurable improvements in test-ordering behaviour, particularly for high-frequency investigations, while also underscoring the need for more tailored approaches for clinically variable tests.

Future directions

Randomised controlled trials should be conducted to assess the long-term sustainability of adherence improvements beyond 6-12 months, quantify cost savings, such as reduced reagent use, and evaluate patient-centred outcomes, including phlebotomy-associated anemia. In addition, given the observed influence of system-level factors on test-ordering behaviour, future interventions should combine educational strategies with workflow and electronic system improvements. Developing institute-specific MRTI guidelines for all laboratory tests and integrating them into the test-ordering platform may help standardise practice and sustain appropriate utilisation.

## Conclusions

A structured educational intervention incorporating MRTI guidance significantly improved adherence to recommended laboratory testing practices, particularly for frequently ordered tests such as CBC, renal function tests, and electrolyte tests. The lack of significant improvement in liver function test ordering highlights the influence of clinical variability and the need for test-specific strategies. Importantly, although the number of tests per patient decreased, the number of tests per patient-day remained comparable, indicating that the intensity of monitoring during hospitalisation was maintained and supporting the safety of the intervention. Although modest in magnitude, the effect is clinically meaningful in high-volume settings and scalable in resource-constrained teaching hospitals. Further studies using controlled designs and system-level interventions are needed to enhance sustainability and generalisability.
